# Unconventional Oil and Gas Energy Systems: An Unidentified Hotspot of Antimicrobial Resistance?

**DOI:** 10.3389/fmicb.2019.02392

**Published:** 2019-10-18

**Authors:** Maria Fernanda Campa, Amy K. Wolfe, Stephen M. Techtmann, Ann-Marie Harik, Terry C. Hazen

**Affiliations:** ^1^Bredesen Center for Interdisciplinary Research and Graduate Education, University of Tennessee, Knoxville, Knoxville, TN, United States; ^2^Biosciences Division, Oak Ridge National Laboratory, Oak Ridge, TN, United States; ^3^Institute for a Secure and Sustainable Environment, University of Tennessee, Knoxville, TN, United States; ^4^Environmental Science Division, Oak Ridge National Laboratory, Oak Ridge, TN, United States; ^5^Department of Biological Sciences, Michigan Technological University, Houghton, MI, United States; ^6^Departments of Civil and Environmental Engineering, Earth and Planetary Sciences, Microbiology, University of Tennessee, Knoxville, Knoxville, TN, United States

**Keywords:** antimicrobial resistance, biocides, hydraulic fracturing, unconventional oil and gas, biological risk, resistome, resistome risk

## Abstract

Biocides used in unconventional oil and gas (UOG) practices, such as hydraulic fracturing, control microbial growth. Unwanted microbial growth can cause gas souring, pipeline clogging, and microbial-induced corrosion of equipment and transportation pipes. However, optimizing biocide use has not been a priority. Moreover, biocide efficacy has been questioned because microbial surveys show an active microbial community in hydraulic fracturing produced and flowback water. Hydraulic fracturing produced and flowback water increases risks to surface aquifers and rivers/lakes near the UOG operations compared with conventional oil and gas operations. While some biocides and biocide degradation products have been highlighted as chemicals of concern because of their toxicity to humans and the environment, the selective antimicrobial pressure they cause has not been considered seriously. This perspective article aims to promote research to determine if antimicrobial pressure in these systems is cause for concern. UOG practices could potentially create antimicrobial resistance hotspots under-appreciated in the literature, practice, and regulation arena, hotspots that should not be ignored. The article is distinctive in discussing antimicrobial resistance risks associated with UOG biocides from a *biological risk*, not a chemical toxicology, perspective. We outline potential risks and highlight important knowledge gaps that need to be addressed to properly incorporate antimicrobial resistance emergence and selection into UOG environmental and health risk assessments.

## Introduction

Unconventional oil and gas (UOG) extraction may create underappreciated hotspots for antimicrobial resistance (AMR). Antimicrobial agents, specifically biocides, are used in UOG extraction to mitigate microbially induced corrosion and gas souring. Conventional oil and gas practices extract hydrocarbon from high-permeability, highly pressurized strata such as limestone. In contrast, unconventional oil and gas (UOG) practices extract hydrocarbon from low permeability strata such as shale using hydraulic fracturing (HF) coupled with horizontal drilling.

HF uses injection fluid containing water, sand, and chemicals. The volume of HF injection fluid can exceed 10 million liters per well, using more than 500 mg/L of biocide (Kahrilas et al., 2014). Typically, 30–90% of the injection fluid does not resurface; fluid that does is referred to as “produced” and “flowback” water (PFW). Injection and PFW create environmental exposure to biocides, and importantly, to microbes causing potential resistance to the biocide (Cluff et al., 2014).

UOG biocides are used alone and in combination. Table 1 summarizes common UOG biocides, their known antimicrobial mechanisms, and the mechanisms’ specificity. While biodegradation and biotransformation are important microbial responses to biocides, they are beyond the scope of this perspective. Differences in modes of action among biocides may trigger different resistance mechanisms in bacteria, which may cause different selective pressures in the environment. While the microbial dynamics, mobility, degradation, physiochemical characteristics, and toxicity of UOG biocides have been discussed before (Gaspar et al., 2014; Kahrilas et al., 2014; Stringfellow et al., 2014), their environmental and public health implications have been ignored.

**Table 1:** Frequently used hydraulic fracturing biocides, their reported mode of action, microbial resistance response, and specificity of the response.

Biocide name and Cas No.	Chemical formula	Frequency of use	Biocide mode of action	Microbial genetic resistance response	Is the known genetic response biocide specific?
Glutaraldehyde 111-30-8	C_5_H_8_O_2_	27%	Electrophilic	Efflux pumps (Vikram et al., 2014; Vikram et al., 2015) Heat-shock like response (Lee et al., 2010)	Efflux pumps confer broad non-specific resistance
Dibromo-nitrilopropionamide 10,222-01-2	C_3_H_2_Br_2_N_2_O	24%	Electrophilic	Not known	N/A
Tetrakis hydroxymethyl phosphonium sulfate 55566-30-88	[(HOCH_2_)_4_P]_2_SO_4_	9%	Electrophilic	Not clear (Lee et al., 2010)	N/A
Didecyl dimethyl ammonium chloride 7173-51-5	C_22_H_48_NCl	8%	Lytic	*qacA* and homologs *qacB-H/J/Z,* and other multidrug efflux pumps (Jennings et al., 2015)	QAC resistance genes are commonly found on plasmids with other multi-drug-resistance genes (Jennings et al., 2015)
Chlorine dioxide 10049-04-4	ClO_2_	8%	Oxidizing	σ^B^, CtsR, and HrcA (Pleitner et al., 2014)	Not known
Tributyl tetradecyl phosphonium chloride 81741-28-8	C_26_H_56_PCl	4%	Lytic	*qacA* and homologs *qacB-H/J/Z,* and other multidrug efflux pumps (Jennings et al., 2015)	QAC resistance genes are commonly found on plasmids with other multi-drug-resistance genes (Jennings et al., 2015)
Alkyl dimethyl benzyl ammonium chloride 68424-85-1	C_19_H_34_NCl	3%	Lytic	*qacA* and homologs *qacB-H/J/Z,* and other multidrug efflux pumps (Jennings et al., 2015)	QAC resistance genes are commonly found on plasmids with other multi-drug-resistance genes (Jennings et al., 2015)
Methylisothiazolinone 2682-20-4	C_4_H_5_NOS	3%	Electrophilic	RND efflux pumps (Rushton et al., 2013)	Efflux pumps confer broad non-specific resistance
Chloro-methylisothiazolinone 26172-55-4	C_4_H_4_NOSCl	3%	Electrophilic	RND efflux pumps (Rushton et al., 2013)	Efflux pumps confer broad non-specific resistance
Sodium Hypochlorite 7681-52-9	NaClO	3%	Oxidizing	*ohr, ahpC, and ahpF,* ROS response (Small et al., 2007)	These genes confer resistance to oxidative stress (Small et al., 2007)
Dazomet 533-74-4	C_5_H_10_N_2_S_2_	2%	Electrophilic	Not known	N/A
Dimethyloxazolidine 51200-87-4	C_5_H_11_NO	2%	Electrophilic	Not known	N/A
Trimethyloxazolidine 75673-43-7	C_6_H_14_NO	2%	Electrophilic	Not known	N/A
N-Bromosuccinimide 128-08-5	C_4_H_4_BrNO_2_	1%	Electrophilic	Not known	N/A
Bronopol 52-51-7	C_3_H_6_BrNO_4_	<1%	Electrophilic	*Rpos* (MacLehose et al., 2004)	No, *rpos* also has a role in antibiotic resistance (Hall et al., 2018)
Peracetic acid 79-21-0	C_2_H_4_O_3_	<1%	Oxidizing	Tetracyclines ARG (Biswal et al., 2014)	Not clear

*Expanded from Kahrilas et al., 2014*.

Currently, we do not know the resistance mechanism for many commonly used UOG biocides, information essential to assess if resistance emergence and selection (1) is biocide-specific or (2) could co-select for a broad spectrum of antimicrobials and antibiotics. Filling these knowledge gaps is critical, as resistance to common antibiotics is becoming increasingly common, leaving many populations vulnerable to complications by common infection. Addressing these knowledge gaps may help prevent or remediate potential AMR risks.

## Antimicrobial Resistance Selective Pressure of Biocides

Stringfellow et al. (2014) reviewed the physical, chemical, and biological characteristics of chemicals used in HF fluids, concluding that the chemical category “biocides” contained the most toxic compounds, with high human and environmental toxicity. In addition to their direct toxicity, sub-lethal concentrations of biocides can indirectly affect human and environmental health. This selective pressure may serve as breeding environments for antimicrobial resistant bacteria (ARB) and, subsequently, antimicrobial resistant genes (ARG) (Russell, 2000, 2003a; Fraise, 2002; Walsh et al., 2003; Davin-Regli and Pages, 2012) that spread AMR in the environment affecting the environmental resistome.

The environmental resistome refers to all of the ARG in an environment carried by bacteria and mobile genetic elements (Wright, 2007). ARG may provide antimicrobial agent-specific resistance or confer nonspecific resistance *via* multidrug efflux pumps. ARG can be part of the microbial core genome or embedded in mobile elements such as plasmids, transposons, and integrons. Mobile elements may carry resistance to specific antimicrobial agents and be transferred to other microbes through horizontal gene transfer (HGT). The non-specificity of the multidrug efflux pumps makes them particularly relevant for antibiotic-biocide cross-resistance (Russell, 2003b). Environmental exposure to subinhibitory concentrations of the antibiotic tetracycline of 10 μg/L, 150 times below the minimal inhibitory concentration, was enough to drive HGT of antibiotic resistance (Jutkina et al., 2016). Unfortunately, data are not available on the minimal concentrations needed to drive HGT for common UOG biocides, nor how physiochemical conditions associated with UOG, such as high salt and organic compounds, may enhance selective pressure for AMR.

Selective pressures that drive ARB and ARG HGT can create a “hotspot” for resistance. It is essential to identify, monitor, remediate, and, if possible, prevent these hotspots to stop the spread of AMR. AMR is currently one of the biggest threats to public and environmental health. While AMR can be naturally occurring (D’costa et al., 2006; Wright, 2007; Allen et al., 2009, 2010; Davies and Davies, 2010), anthropogenic stresses are the most significant selective pressures in antimicrobial hotspots (D’costa et al., 2006). Anthropogenic hotspots from pharmaceutical, agricultural, and municipal wastewater industries have garnered much attention (Rizzo et al., 2013; Fair and Tor, 2014; Woolhouse et al., 2015). UOG could be an underappreciated anthropogenic hotspot that should be studied further.

## Fate and Transport Studies of Unconventional Oil and Gas Biocides Missing Antimicrobial Resistance Selective Pressure Risk; Potential Routes of Exposure

**Figure 1:**
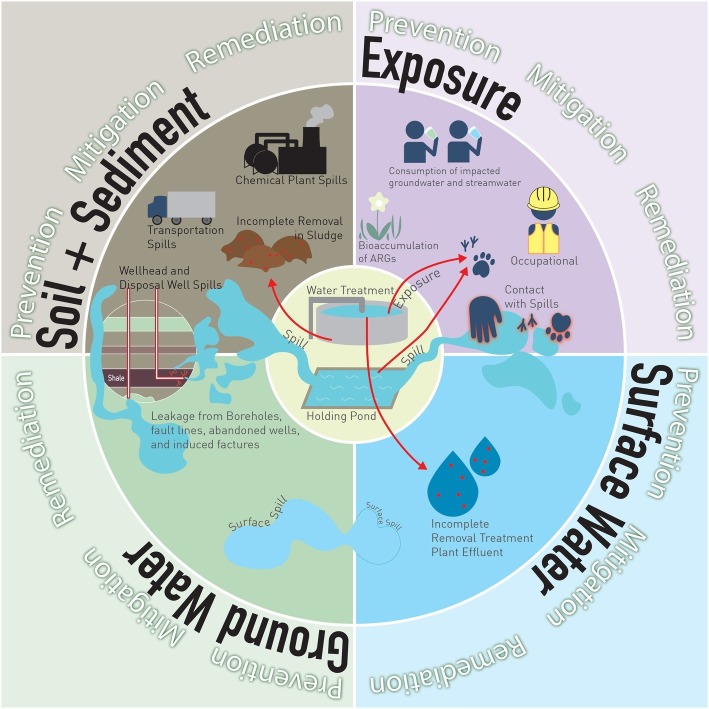
Potential sources of biocide release and ARB and ARG enrichment and exposure. *Release. To soil, from* (1) transportation spills, (2) chemical plant spills, (3) holding pond spills, (4) wellhead spills, (5) disposal well spills, (6) incomplete removal in treatment plants, sludge applied to agricultural top-soil. *To surface water, from* (7) surface spill runoff, (8) incomplete removal in a treatment plant, effluent disposed of in streams. *To shallow groundwater, from* (9) surface spills leaching into shallow aquifers, (10) borehole leakage, fault lines, and abandoned wells, (11) induced fractures. *Exposure. By animals, from contact with* (12) holding ponds, (13) effluent from treatment plants, (14) spills. *By humans, from* (15) contact with spills, (16) consumption of affected stream water, (17) consumption of affected groundwater.

UOG biocides can be released in the environment through spills, incomplete removal of biocides in wastewater treatment, and reuse/repurpose of PFW (Figure 1). Data on UOG spills are highly variable because of the diversity of state laws, and because disclosure responsibility falls on well operators or owners. Annual spill rates of up to 15% have been reported in high-UOG activity states (Patterson et al., 2017). Variability in reporting and local regulations may also limit the capability of companies or states to intervene and promptly clean spills, perhaps contributing to incomplete removal of biocides. Although incomplete removal of antimicrobials and, subsequently, ARB and ARG in wastewater treatment facilities (and other bio-based economies such as agriculture) are topics of intense discussion (Rizzo et al., 2013), they are beyond the scope of this article. To decrease reliance on local water resources, some operators reuse PFW to fracture new wells (Chen and Carter, 2016), and some states allow PFW re-use for road salting (Skalak et al., 2014) and agriculture (Stringfellow et al., 2017). Such activities may further expand the reach of biocide-induced AMR in the environmental resistome.

Once released to the environment, biocides can affect or be transported through soil, surface water, or groundwater, with varied pathways for animal and human exposure (Figure 1). Biocides present in HF fluids downhole have a different fate than biocides in surface spills, due to chemical interactions, temperature, pressure, and oxygen fluctuations downhole. Only one study has explored the fate and transport of a biocide downhole. The study showed that glutaraldehyde, the most commonly used UOG biocide, has limited antimicrobial activity in an alkaline and/or hot formations, but it is more persistent in lower temperatures, higher acidity and/or salinity (Kahrilas et al., 2016). Glutaraldehyde also polymerizes at different high temperatures and salinities found in shales. The polymerization of glutaraldehyde affects its biocidal properties by altering the number of available crosslinking sites (Migneault et al., 2004; Wine et al., 2007). However, this study did not investigate the efficacy of biocides under downhole conditions.

There are indications that subsurface microorganisms confer high incidence of AMR and that they harbor high concentrations of mobile genetic elements such as plasmids (Fredrickson et al., 1988; Brown and Balkwill, 2008). The difference between surface and subsurface microbial communities is important, as biocide efficacy tests are based on different conditions from those encountered downhole and employ lab strains of bacteria whose physiologies inaccurately represent subsurface bacteria. Biocides’ surface spills would have a different fate than in the subsurface. For example, the absorption of glutaraldehyde to soil decreases its reactive availability and biocidal efficiency (McLaughlin et al., 2016). Glutaraldehyde can also be more persistent in areas previously affected by UOG activity (Campa et al., 2018). It is unclear if limited biocidal efficiency still can select for ARB. However, even low concentrations of biocides (~5 mg/L) caused microbial inhibition, presumably capable of causing selective pressure (Rogers et al., 2017).

Fate and transport of other biocides have been studied for some industries, but not in the context of UOG activity. However, Kahrilas et al. (2014) reviewed the available literature to predict their fate in UOG conditions, for example, quaternary ammonium compounds (QACs) biocides are ubiquitous in domestic and industrial products. QACs tend to accumulate in wastewater treatment plants and are introduced in the environment as plant effluent or sludge (Zhang et al., 2015). Once in the environment, QACs can either degrade or undergo absorption with the potential for leaching. Sorption occurs more quickly than degradation. Protracted microbial exposure to even sub-inhibitory concentrations of QACs was shown to select for resistance to clinically relevant antibiotics (Tandukar et al., 2013; Zhang et al., 2015; Mulder et al., 2018). QACs have been detected in PFW, indicating that they are not fully depleted or transformed downhole (Ferrer and Thurman, 2015). Similar studies are needed for all common UOG biocides to determine the risk imposed by the biocides’ selective pressure.

The spatial-temporal dimensionality of UOG biocide and related AMR mobility have not been explored sufficiently to establish a causal link between UOG biocide usage and health and environmental risks in population living near the wells. However, communities living within a 1 km radius of an UOG well are more likely to report dermal and upper respiratory symptoms than populations between 1 and 2 km (Rabinowitz et al., 2015). In 2010, an estimated 17.6 million people in the US lived within 1.6 km of an active oil and gas well (conventional and/or unconventional) (Czolowski et al., 2017), without including exposure from disposal facilities. The UOG biocide related AMR risk needs to be quantified for these populations.

## Antimicrobial Resistance Enrichment Potential in the Environment

Direct toxicity studies of biocides used in UOG do not provide a systematic measure of ARB enrichment potential. Literature discussing potential risks focuses on water availability (Jiang et al., 2014; Absar et al., 2018), and water contamination (Osborn et al., 2011; Akob et al., 2016; Ulrich et al., 2018). Little attention has been paid to the implications of UOG biocides for the environmental microbiome and resistome. Environmental surveys associated with UOG have mostly focused on the microbial phylogeny of pre-and post-production water (Mouser et al., 2016) or of impacted sites (Trexler et al., 2014; Chen See et al., 2018; Ulrich et al., 2018). Few studies focus on the functional potential of PFW microbiome (Mohan et al., 2014; Daly et al., 2016) and even fewer on the metagenomic potential of UOG-impacted sites microbiome (Fahrenfeld et al., 2017). Regardless of biocide usage and the harsh/extreme conditions present downhole, microbes were active and present in the PFW (Struchtemeyer and Elshahed, 2012; Daly et al., 2016; Vikram et al., 2016). Microbial presence is evidence that neither conditions nor biocide dosages were sufficient to completely inhibit bacteria (Struchtemeyer et al., 2012), but conditions and dosages do alter microbial community composition (Murali Mohan et al., 2013). Although biocides do not completely inhibit their targets, they trigger microbial defense mechanisms (Gaspar et al., 2014; Kahrilas et al., 2014) that facilitate ARB enrichment and ARG propagation.

UOG wastewater disposal facilities can impact nearby aquifers, altering microbial community composition (Akob et al., 2016). Most of the orders detected by Akob et al. (2016) were inconspicuous. However, some orders, such as *Clostridiales*, include pathogenetic species, making ARG HGT in these bacteria a higher risk. *Clostridiales* are members of the *Fimicutes* phylum, the most frequent carriers of acquired ARG (Berendonk et al., 2015). Furthermore, studies show that known pathways of AMR such as stress response, sporulation, dormancy, and efflux pumps are present or upregulated in UOG wastewater (Mohan et al., 2014; Vikram et al., 2016). Surface water affected by UOG activities also contains genetic markers for AMR, efflux pumps, and dormancy, all associated with stress caused by biocides (Fahrenfeld et al., 2017). The detection of these genomic markers of AMR is another indication of the existing risk.

Microbial metagenomic analyses of those same UOG-impacted aquifers revealed detection of ARG (Fahrenfeld et al., 2017). Most of the enriched genes coded for multidrug efflux pumps. Multidrug efflux pumps have been identified as enriched in the presence of UOG biocides (Vikram et al., 2015). Fahrenfeld et al. (2017) compared the profiles and quantities of ARG found in UOG-impacted streams to municipal wastewater that was deemed a hotspot of AMR and to an anthropogenically impacted salt river (Fahrenfeld et al., 2017). More ARG types were detected in the UOG-impacted stream than the impacted salt river, but the concentrations of ARG (ppm) were comparable across sites. Nevertheless, municipal wastewater contained more ARG types and generally higher ppm levels of ARG. Currently there is no consensus on “acceptable” levels of ARG in wastewater treatment plants or aquifers (Rizzo et al., 2013). To address the possibility of an AMR hotspot forming, ARG quantification could be part of ecological monitoring efforts in high-density UOG areas to allow early detection of ARG spreading or increasing in abundance.

We have recently showed that streams impacted by UOG spills containing glutaraldehyde are more resistant to the biocide when exposed to it again as compared to pristine streams (Campa et al., 2018). However, the resistance was biocide-specific, as DBNPA did not show enhanced resistance in the same streams compared to pristine streams (Campa et al., 2019). More studies providing clear evidence of environmental resistance and biocide-biocide and biocide-antibiotic cross resistance are needed to determine which biocides minimize health and environmental risk.

Multidrug efflux pumps, especially the AcrAB-MexAB family, frequently are reported in human infectious diseases (Nikaido and Pagès, 2012). Those efflux pumps have been detected at high levels in UOG-impacted areas and reported as mechanisms for resistance for glutaraldehyde. However, a method to distinguish between core resistance genes within the microbial community and mobile genetic elements due to selective pressure by UOG biocides is unclear.

Not all ARG in the resistome pose the same risk (Martínez et al., 2014). ARG that are part of the general genome of specific microbial taxa pose less risk than ARG located within mobile genetic elements, which have a higher probability of being horizontally transferred to other bacteria. Martínez et al. (2014) outlined seven resistance readiness conditions based on identity, demonstrated functional evaluation, and mobility (Martínez et al., 2014). Bengtsson-Palme and Larsson (2015) argued that Martinez et al. downplayed the risk caused by mobile genetic elements, even though they have not been detected in human pathogens, which may be the case in UOG biocide caused ARG.

## Framework to Fill Knowledge Gaps Surrounding Unconventional Oil and Gas Related Antimicrobial Resistance Risk to Humans and the Environment

To address AMR-related risks in UOG-impacted ecosystems, we suggest a framework that quantifies risk based on metagenomic evaluation and mobility, while also incorporating potential threats from unknown ARG and mobile genetic elements. For example, AcrAB-MexAB efflux pumps would be categorized as low-level risk based on the Martínez et al. (2014) risk rating scheme, as their resistance is not antibiotic specific. However, to fully characterize their risk, determining if the pumps are in mobile genetic elements would be needed, or if they are in high abundance because of the taxa enriched due to other geochemical parameters of UOG wastewater. By understanding if the ARG genes are in mobile genetic elements, we could better understand the range of risks for UOG workers and impacted communities.

Known biocide-associated ARG can be detected and quantified using high throughput (HT) qPCR and metagenomics surveys. HT-qPCR has the advantage of being more sensitive and requiring less DNA input than metagenomics, although ARG of interest need to be known prior to analyses for primer design. In contrast, metagenomics have the advantage of analyzing unknown sequences that may be related to novel ARG-associated genes or mutations of known genes (Waseem et al., 2019). Databases such as CARD are good resources to detect ARG and potentially novel ARG from metagenomic surveys (Jia et al., 2017). Metagenomics has been proposed as a tool for environmental monitoring and may provide early warning for environmental impacts of anthropogenic activity (Hazen et al., 2013; Techtmann and Hazen, 2016). However, caution needs to be exercised since all genomic analyses have limitations that may bias conclusions according to sample collection, nucleic acid extraction, processing, sequencing platforms, and Chip technologies (Hazen et al., 2013).

Using Kahrilas et al. (2014) list of frequently used biocides according to FracFocus.org, we compiled the reported microbial genetic responses to common UOG biocides (Table 1). We found that 6 out of 16 biocides do not have a reported microbial genetic resistance mechanism. The 10 biocides that do seem to confer broad resistance to other antimicrobials and antibiotics. Resistance to QAC biocides seems to be carried in mobile genetic elements carrying other resistance genes (Jennings et al., 2015), and it remains unclear if the resistance responses for other biocides are also evident in mobile genetic elements. This information could be gathered from mobile genetic element-specific primers or through metagenomic surveys.

## Conclusion

While the current risk of UOG-associated AMR is not known, it should be taken into account when considering the environmental impacts of UOG. AMR carries a known, serious category of risks. UOG biocide-related practices create potential risks that extend far beyond “occupational hazards.” Spills and accidental release of biocides expose soil, surface water, and groundwater, allowing ARB and ARG to reach environmental niches beyond UOG wells or holding ponds. Using PFW for irrigation, road salting, or other purposes could contribute to the unintended enrichment and propagation of AMR.

Current UOG risk assessments focus on the toxicology of HF chemicals and not the biological risk of AMR. Standardized risk assessment field tests are needed to understand and quantify the potential risks of bacterial resistance, and to better predict the stochastic occurrence and spatial-temporal spread of AMR. Conducting such studies is challenging because access to UOG areas is limited, spills may go unreported, and the regulation around UOG varies by state/country.

We need to develop a better understanding of the microbial resistance mechanisms to common UOG biocides and their impacts on the environmental resistome. When spills occur, HT-qPCR of known ARG and metagenomic surveys [following reproducible, robust, and open science methodologies such as the one suggested by phase two of the Earth Microbiome Project (Thompson et al., 2017)] should be conducted alongside traditional chemical assessments to facilitate comparison studies. HT-qPCR would help quantify ARG of interest in affected areas and monitor their change over time while metagenomics would help to identify new ARG of interest. qPCR and metagenomic surveys should discuss if the ARG is present in mobile elements and if it is detected across different taxa (horizontal vs. vertical ARG enrichment). Post-spill metagenomics should be compared to upstream of background areas. These metagenomic monitoring efforts will help confirm if the spill caused an increase in ARG and the origin of ARG. We also echo others’ calls for ARG to be treated as micropollutants and to establish a maximum ARG concentration that is deemed acceptable, based on its risk to human and environmental health (Berendonk et al., 2015).

Lastly, we hope attention to UOG-related AMR risks will influence HF practices. Optimizing biocide usage may decrease the concentrations of biocides needed to achieve desired effects, thereby preventing and/or reducing adverse human health and environmental consequences. Other methods of bacterial control, such as competitive exclusion, may be more successful from combined business, environmental, and human health perspectives.

## Data Availability Statement

The datasets generated for this study are available on request to the corresponding author.

## Author Contributions

MC and AW wrote the manuscript with discussions, reviews, and input from TH, ST, and A-MH. A-MH prepared the figure.

### Conflict of Interest

The authors declare that the research was conducted in the absence of any commercial or financial relationships that could be construed as a potential conflict of interest.

## Abbreviations

AMR: Antimicrobial resistance
ARB: Antimicrobial resistant bacteria
ARG: Antimicrobial resistant genes
HF: Hydraulic fracturing
HGT: Horizontal gene transfer
PFW: Produced and flowback water
QAC: Quaternary ammonium compounds
UOG: Unconventional oil and gas
